# DECISIONS, DECISIONS: CHARACTERIZING WORKERS' DAILY DECISION PROCESSES DURING LEISURE TIME

**DOI:** 10.1093/geroni/igac059.051

**Published:** 2022-12-20

**Authors:** Claire Smith, Soomi Lee

**Affiliations:** University of South Florida, Tampa, Florida, United States; University of South Florida, Tampa, Florida, United States

## Abstract

Decisions during adulthood set the foundation for healthy aging, but descriptions of healthy and unhealthy decision processes are missing. We extracted latent profiles of daily decision resources (energy and affect) and linked them to daily leisure activity. Diary data was collected from working adults (N=83; Mage=37 years) over the ten workdays (N=693). We identified three daily decision profiles consistent with the Decision Triangle – (1) logical (energetic, unemotional), (2) automatic (less energetic, unemotional), and (3) visceral (unenergetic, highly emotional) – and one additional profile, (4) mild visceral (moderately unenergetic, moderately emotional). Daily logical decision-making related to more “want” leisure activities (i.e., aligned with desires/interests) and the greatest variety in leisure activities. Automatic engaged in the most chores. Visceral engaged in the fewest social activities and least variety in leisure activities. Our findings advance understanding of specific decision processes during leisure, which may have consequences for health and well-being as a person ages.

